# Quality of care in elder emergency department patients with pneumonia: a prospective cohort study

**DOI:** 10.1186/1471-227X-8-6

**Published:** 2008-04-30

**Authors:** Jeffrey M Caterino, Brian C Hiestand, Daniel R Martin

**Affiliations:** 1 Department of Emergency Medicine, The Ohio State University, Means Hall, 1654 Upham Drive, Columbus, OH 43210, USA

## Abstract

**Background:**

The goals of the study were to assess the relationship between age and processes of care in emergency department (ED) patients admitted with pneumonia and to identify independent predictors of failure to meet recommended quality care measures.

**Methods:**

This was a prospective cohort study of a pre-existing database undertaken at a university hospital ED in the Midwest. ED patients ≥18 years of age requiring admission for pneumonia, with no documented use of antibiotics in the 24 hours prior to ED presentation were included. Compliance with Pneumonia National Quality Measures was assessed including ED antibiotic administration, antibiotics within 4 hours, oxygenation assessment, and obtaining of blood cultures. Odds ratios were calculated for elders and non-elders. Logistic regression was used to identify independent predictors of process failure.

**Results:**

One thousand, three hundred seventy patients met inclusion criteria, of which 560 were aged ≥65 years. In multiple variable logistic regression analysis, age ≥65 years was independently associated with receiving antibiotics in the ED (odds ratio [OR] = 2.03, 95% CI 1.28–3.21) and assessment of oxygenation (OR = 2.10, 95% CI, 1.18–3.32). Age had no significant impact on odds of receiving antibiotics within four hours of presentation (OR 1.10, 95% CI 0.84–1.43) or having blood cultures drawn (OR 1.02, 95%CI 0.78–1.32). Certain other patient characteristics were also independently associated with process failure.

**Conclusion:**

Elderly patients admitted from the ED with pneumonia are more likely to receive antibiotics while in the ED and to have oxygenation assessed in the ED than younger patients. The independent association of certain patient characteristics with process failure provides an opportunity to further increase compliance with recommended quality measures in admitted patients diagnosed with pneumonia.

## Background

Over 900,000 patients aged ≥65 years develop pneumonia each year in the United States (U.S.) [1,2]. This results in over 600,000 hospitalizations and 57,000 deaths per year in the U.S. elderly population, making pneumonia the 5^th ^leading cause of death among U.S. elders[1,3,4]. The majority of elderly patients admitted with a diagnosis of pneumonia enter through the emergency department (ED)[5]. In 2003 alone, an estimated 662,000 elder patients were diagnosed with pneumonia in U.S. EDs [6].

In an attempt to decrease pneumonia-related morbidity and mortality, the Joint Commission on Accreditation of Healthcare Organizations (JCAHO) and the Centers for Medicare & Medicaid Services (CMS) have established a series of evidence-based quality measures for patients admitted with pneumonia. The measures include: administration of appropriate antibiotics within four hours of presentation, oxygenation assessment within 24 hours of arrival, collection of blood cultures within 24 hours in patients who are admitted or transferred to the intensive care unit (and before antibiotic administration if cultures are obtained in the ED), appropriate screening for influenza and pneumococcal vaccination, and smoking cessation counseling [7]. Given the time sensitive nature of many of these measures, their completion in the ED is necessary to satisfy the current standards.

Studies have demonstrated that not all ED patient subpopulations receive recommended care processes at the same rates as the general ED population. For example, racial disparities have been demonstrated in care provided to ED patients with pneumonia [8]. In other conditions such as acute myocardial infarction it has been demonstrated that disparities in ED care related to patient age exist [9], and it is reasonable to consider that a similar disparity might exist in the treatment of pneumonia. Elders with infection often demonstrate a relatively nonspecific clinical presentation and such presentations have been associated with delays in care [10]. Thus, the possibility exists that elders with pneumonia do not receive quality care at the same rate as younger patients.

In elders admitted with pneumonia, previous studies have identified hospital and patient factors in the elderly which predict non-adherence to processes of care [10,11]. However, none have compared the rates of adherence in elders to that of younger patients in the ED. The primary aims of this study were to determine relative adherence to proven process of care measurements and to identify factors contributing to suboptimal process implementation in elderly ED patients admitted with pneumonia. We hypothesized that study patients ≥65 years old would be less likely than those under the age of 65 to (1) receive antibiotics in the ED, (2) receive antibiotics within 4 hours, (3) undergo oxygenation assessment in the ED, and (4) have blood cultures obtained in the ED. We also hypothesized that we would be able to identify factors which were independently associated with process failure.

## Methods

### Study design, setting, and population

We conducted a non-concurrent prospective cohort study of a pre-existing database. Database patients were admitted from the ED of an urban, academic tertiary care center with a diagnosis of pneumonia between 1/1/2001 and 6/30/2005. All admitted ED patients were screened for database inclusion. Both the database and this study were approved by the hospital's Institutional Review Board as was a waiver of informed consent.

Potential subjects for the database were identified by review of the daily ED census with confirmation of eligibility by review of ED and inpatient charts. Database inclusion criteria included age ≥ 18 years, hospital admission, and an admitting diagnosis of pneumonia by the ED physician as documented in the medical record. Only admitted patients were considered as the quality measures were studying are designed to be applied only to inpatients. Given that measurement of compliance with quality measures is based on the presence of a physician diagnosis, all patients diagnosed with pneumonia by the ED physician were included in the database.

Patients were excluded from the original database if they received no diagnosis of pneumonia in the ED, were incarcerated, or were seen primarily by the major trauma response team. Patients who had received documented pre-ED antibiotics were excluded from this study's primary data analyses for several reasons. Primarily, we sought to minimize confounding that could occur due to prior antibiotic treatment. While ordinarily such potential confounders would simply be included in the analysis at hand, in this setting patients who have received antibiotics prior to presentation are excluded from the JCAHO and CMS quality assessment programs. As our study outcomes are derived from these quality assessment programs, we elected to mirror their exclusion requirements. A similar approach has been followed in previous studies [8,12].

### Data collection

Data was initially abstracted from paper and computerized medical records. Abstraction was performed by trained data abstractors (research associates and medical students) using a standardized abstraction form. An instruction book was available and all abstractors attended a training session on the content and coding of each data element. Data abstractors attended monthly meetings to resolve any questions and review coding rules. Abstractors were not aware at the time of abstraction of the specific hypotheses evaluated in this study.

To ensure the reliability of the data collection process a data manager monitored day-to-day collection and resolved questions. After study subjects were enrolled, the data manager verified eligibility in a sample of patients, confirmed the accuracy of the data and reviewed charts to locate missing data. The data was then entered into an Excel database. Missing data in categorical variables was assumed to be not present, negative, or not performed, depending on the context of the variable. Patients with missing continuous variables were excluded from the regressions. In cases of conflict, physician notes were considered as more accurate than nursing.

### Definition of variables

Abstracted variables included demographics, ED vital signs, receipt of pre-ED antibiotics, patient symptoms, and presence of co-morbid conditions. Antibiotic administration in the ED and time from triage to initiation of antibiotic was recorded, as were the collection of blood cultures and any assessment of oxygenation, whether pulse oximetry or arterial blood gas analysis. Based on documented ED physician findings, lung exam was coded as normal/abnormal. The exam was considered abnormal in the presence of any of the following: rhonchi, rales, wheezes, decreased breath sounds, or egophony. Laboratory and radiographic results from ED testing were recorded. Attending radiologist chest x-ray results from the ED studied were coded as consistent/inconsistent with pneumonia. Mention by the radiologist of infiltrate, consolidation, air space disease, or pneumonia was considered positive for pneumonia. Mention of only atelectasis was considered negative for pneumonia.

### Outcome measures

The primary independent variable of interest for this study was age ≥ 65 years at time of ED visit. The primary dependent variables included administration of antibiotics in the ED, administration of antibiotics within 4 hours of ED arrival, blood cultures performed in the ED, and documented assessment of oxygenation in the ED. We focused on these processes as they are relevant to ED care of the patient. We excluded vaccination screening and smoking cessation counseling as these are not performed in the ED. Given the inherent difficulty in retrospectively determining appropriateness of antibiotic selection, this measure was also excluded. Such an analysis would have required assessment of multiple factors not available in the database including: recent antibiotic use, the presence of healthcare-associated pneumonia, the administration of additional antibiotics after admission, drug allergies, immunosuppression, concurrent illnesses, and causative organisms.

Patients were considered to have received antibiotics in the ED if they had a charted antibiotic delivered as noted in the ED nursing notes. Patients were considered to have received antibiotics within 4 hours of ED arrival if they had initiation of any antibiotic within 4 hours of triage time in the ED which represents most closely the actual time of patient arrival. Blood cultures were considered performed in the ED if ordered by the ED physician and were either documented as obtained in the ED or were demonstrated as received in the hospital's microbiology database. Blood cultures that were ordered but either not documented as obtained or not clearly received in the lab while the patient was still in the ED were considered as not obtained in the ED. Oxygenation was considered as assessed by documentation either of pulse oximetry or arterial blood gas analysis in the ED. In all cases, absence of documentation was coded as if that process was not performed in the ED.

### Data analysis

In each age group (<65 and ≥65 years old) patient characteristics and the proportion of patients receiving each process of care were noted. Additionally, the proportion receiving each process of care was analyzed by decade over the age of 65. Descriptive statistics included proportions and means with confidence intervals. The T-test and Fisher's exact test were used to evaluate continuous and categorical data, respectively. To assess interrater reliability, a random sample of 21 charts as coded by abstractors was compared with a standard review by the primary author and kappa statistics calculated.

Logistic regression modeling was performed to identify independent predictors of process failure for each process of care. Forward modelling was used to evaluate the contribution of various covariates on the impact of age on each process. Therefore, for each analysis age as a dichotomous variable at 65 years was retained in the regression. Likelihood ratio testing was performed to determine the selection of covariates for evaluation in each model. Those factors with a p < 0.20 in univariate analysis were included progressively into the model until no further additional covariates increased the predictive ability of the model, using p < 0.05 as the cut off for retention in the model. As the database was robust with the number of subjects with outcome failures (lack of blood cultures, antibiotics not administered, oxygenation not assessed) we had the power to include up to 10 covariates into the model, using the standard "rule of 10" for regression sample size determination [13].

Covariates considered in each model included: sex, race (considered as a white/non-white dichotomous variable), presence of co-morbidities, presence of presenting symptoms, initial ED vital signs, lung exam (normal or abnormal), laboratory data, and the presence of pneumonia on chest x-ray. Vital signs and laboratory studies were coded as continuous variables, all others were dichotomous variables. Continuous variables were tested for linearity in the logit of each model by both graphical analysis and the fractional polynomial method [14]; accordingly, variable transformation was not required. Standard regression diagnostics were performed to identify collinearity, test for specification error, and evaluate for highly influential subjects and covariate patterns. No data elements or subject data required deletion from consideration. Biologically plausible interaction terms were generated using the product method and tested for inclusion each model. Age as a dichotomous variable, as previously described, was included in generating interaction terms. As we were not attempting predictive modelling, goodness of fit statistics were not calculated. Analyses were performed using Stata, version 9 (StataCorp LP, College Station, TX).

## Results

There were a total of 2,854 cases abstracted and entered into the database, including 1,942 admitted (68%), 889 discharged (31.1%), and 23 (0.01%) with missing data. In the overall database, 871 patients were ≥65 years old (30.5%). Admission rates were 60.1% for those <65 and 88.0% for elders.

Of the admitted patients, 572 (29.4%) had received pre-ED antibiotics and thus were excluded from the primary analysis. The remaining 1,370 patients were included in the study. Of these, 810 were <65 and 560 were ≥65 years old, including 262 aged 65–74 years, 209 aged 75–84 years, and 89 aged ≥85 years. Mean age was 59.3 years (95% CI, 58.9–60.2). Mean age in those <65 years old was 47.6 years (95% CI, 46.8–48.4) and in those ≥65 years old was 76.2 years (95% CI, 75.6–76.9).

Kappa statistics from a random sample of 21 charts were calculated by comparing results as coded by abstractors as compared with a review by the primary author. The kappa statistic for agreement in administration of pre-ED antibiotics was ĸ = 0.89, for administration of antibiotics in the ED ĸ = 0.85, for a chest x-ray consistent with pneumonia ĸ = 0.52, and for blood cultures drawn in the ED ĸ = 0.74. For past medical history items kappa was 1 for congestive heart failure, renal failure, HIV, and diabetes. Kappa was 0.82 for presence of COPD and 0.64 for presence of liver disease. The kappa statistics for patient symptoms were 0.70 for cough, 0.80 for shortness of breath, and 0.81 for fever or chills. Documented oxygen saturation agreed in 17 of 21 cases and white blood cell count in 17 of 18 cases.

Demographic, medical history, laboratory, and clinical characteristics stratified by age group are noted in Table 1. Elders were more likely to be female and white. They were less likely to complain of classic symptoms of pneumonia such as cough, fever, shortness of breath, or chest pain. However, they were more likely to complain of confusion or altered mental status (20.0% vs. 6.8%). Vital signs and diagnostic findings generally did not differ between the groups except for a lower pulse and a small mean increase in systolic blood pressure and blood urea nitrogen (BUN) in elders. Elders were more likely to have a variety of co-morbidities associated with advancing age such as cancer, heart failure, stroke, COPD, and diabetes.

**Table 1:** Patient Characteristics Stratified by Age Group*

	Entire study population		Patients <65 years old		Patients ≥65 years old	
Patient Characteristics	(n = 1,370)		(n = 810)		(n = 560)	

**Demographics**						
Male sex	56.8%	(54.2–59.5)	60.0%	(56.6–63.4)	52.2%	(48.1–56.4)
White race	66.8%	(64.2–69.3)	60.9%	(57.4–64.2)	75.4%	(71.6–78.9)
						
**Presence of patient complaints**						
Cough	60.4%	(57.8–63.0)	63.8%	(60.5–67.1)	55.4%	(51.2–59.5)
Fever	44.0%	(41.4–46.6)	50.1%	(46.7–53.6)	35.2%	(31.2–39.1)
Sputum production	34.2%	(31.6–36.7)	36.4%	(33.1–39.7)	30.9%	(27.1–34.7)
Shortness of breath	66.9%	(64.4–69.4)	70.4%	(67.2–73.5)	61.8%	(57.7–65.8)
Abdominal pain	6.4%	(5.1–7.6)	7.4%	(5.6–9.2)	4.8%	(3.0–6.6)
Confusion/altered mental status	12.2%	(10.5–13.9)	6.8%	(5.1–8.5)	20.0%	(16.7–23.3)
Chest pain	29.8%	(27.4–32.2)	35.1%	(31.8–38.4)	22.1%	(18.7–25.6)
Nausea	4.5%	(3.4–5.5)	4.7%	(3.2–6.2)	4.1%	(2.5–5.8)
Diarrhea	5.2%	(4.0–6.4)	6.4%	(4.7–8.1)	3.4%	(1.9–4.9)
						
**Presence of co-morbidities**						
Neoplasm	23.1%	(20.9–25.4)	19.4%	(16.7–22.1)	28.6%	(24.8–32.3)
Congestive heart failure	10.9%	(9.2–12.5)	7.3%	(5.5–9.1)	16.1%	(13.0–19.1)
Renal failure	9.3%	(7.8–10.9)	9.3%	(7.3–11.3)	9.5%	(7.0–11.9)
Liver disease	3.6%	(2.6–4.6)	5.6%	(4.0–7.1)	0.7%	(0.0–1.4)
Cerebrovascular disease	8.1%	(6.7–9.5)	5.4%	(3.9–7.0)	12.0%	(9.3–14.7)
HIV/AIDS	5.0%	(3.9–6.2)	8.5%	(6.6–10.4)	0.0%	(0.0–0.0)
Asthma	9.3%	(7.8–10.9)	12.8%	(10.5–15.1)	4.3%	(2.6–6.0)
Steroids	7.7%	(6.3–9.2)	9.0%	(7.0–11.0)	5.9%	(3.9–7.38)
Chronic obstructive pulmonary disease	20.4%	(18.3–22.6)	17.3%	(14.7–19.9)	25.0%	(21.4–28.6)
Diabetes	24.2%	(21.9–26.4)	21.2%	(18.4–24.1)	28.4%	(28.4–32.1)
Organ transplant	1.9%	(1.2–2.6)	2.8%	(1.7–4.0)	0.5%	(0.0–1.1)
						
**Mean initial ED vital signs**						
Temperature (°F)	98.9	(98.8–99.0)	99.0	(98.8–99.1)	98.7	(98.6–98.9)
Systolic blood pressure (mmHg)	129.1	(127.6–130.7)	125.4	(123.5–127.3)	134.5	(132.1–136.9)
Respiratory rate (breaths/minute)	22.8	(22.5–23.2)	23.0	(22.5–23.4)	22.6	(22.1–23.2)
Pulse (bpm)	102.8	(101.6–103.9)	107.5	(106.1–109.0)	95.9	(94.2–97.6)
						
**Diagnostic findings**						
Lung Exam Normal	19.5%	(17.3–21.6)	19.8%	(16.9–22.6)	19.1%	(15.7–22.5)
White blood cell count (cells/mm3)	12.5	(12.2–12.9)	12.5	(12.0–13.0)	12.6	(12.0–13.2)
Sodium (mmol/L)	135.6	(135.4–135.9)	135.3	(134.9–135.7)	136.1	(135.7–136.5)
Blood urea nitrogen (mg/dL)	22.0	(21.1–22.9)	20.0	(18.8–21.3)	24.7	(23.3–26.0)
Creatinine (mg/dL)	1.52	(1.43–1.62)	1.53	(1.38–1.67)	1.52	(1.4–1.6)
Glucose (mg/dL)	138.7	(134.9–142.6)	135.9	(130.6–141.3)	142.6	(137.3–147.9)
						
**Pneumonia severity index**						
1–2	34.4	(31.9–36.9)	51.0	(47.6–54.4)	10.3	(8.1–13.2)
3	21.4	(19.3–23.6)	22.5	(19.4–25.5)	19.8	(16.7–23.3)
4	33.8	(31.3–36.3)	21.7	(19.0–24.7)	51.2	(51.2–55.4)
5	10.4	(8.9–12.2)	4.8	(3.5–6.5)	18.6	(15.6–22.0)

*Categorical variables expressed as n% (95% confidence interval) and continuous variables expressed as mean (95% CI) of those with the characteristic.

°F = degrees fahrenheit, bpm = beats per minute

A total of 1163 (84.9%) study patients had an abnormal chest x-ray as defined by JCAHO criteria. The rates were consistent between elders (84.5%) and younger patients (85.3%). Information was not available in the database on CT scan results which may have led to the diagnosis of pneumonia. Percentage of ED antibiotic administration in those without abnormality on chest x-ray was 89.4% (95% CI, 94.3–93.2) as compared to a total percentage ED antibiotic administration of 91.3% (95% CI, 89.7–92.8).

Initial temperature was ≥100.4°C in only 103 (22.0%) patients 65 and over, and it was ≥99.0°C in only 210 (37.5%). In younger patients, 233 (28.8%) had temperatures ≥100.4°C during their ED stay and 365 (45.1%) had a temperature ≥99.0°C. Mean time to antibiotic administration was 3.21 hours; 3.20 hours in those under 65 and 3.22 hours in those 65 and over (p = 0.846, Student's T test).

A total of 1251 patients received antibiotics in the ED (91.3%), including 724 of 810 younger patients (89.4%) and 527 of 560 patients ≥65 years old (94.1%). Antibiotics were administered within 4 hours in 910 (66.4%) patients, including 529 (65.0%) younger patients and 381 (68.0%) elders. ED blood cultures were obtained in 939 (68.5%) patients, including 558 (68.9%) younger patients and 381 (68%) elders. Oxygenation was assessed in 1302 (95.0%) of patients, including 761 (93.9%) younger patients and 541 (96.6%) elders.

To determine independent associations and variables associated with processes of care, regression models were constructed for each process (Table 2). Age ≥65 years was associated with improved odds of receiving antibiotics while in the ED (OR = 2.03, 95% CI 1.28–3.21), as was triage heart rate. Factors associated with a decreased chance of receiving antibiotics in the ED included the presence of confusion, a complaint of shortness of breath, HIV, or a normal lung exam. No interaction terms contributed significantly to this model. Nine subjects were excluded from this model due to missing heart rate values.

**Table 2:** Patient Characteristics Independently Associated with Each Process of Care*

**Characteristics classified by each process**	**Odds ratio (95% CI)**		**P value**
**Any ED antibiotics**			
Age ≥65	2.03	(1.28–3.21)	0.003
Heart rate†	1.01	(1.004–1.02)	0.005
Confusion	0.48	(0.27–0.85)	0.012
HIV	0.31	(0.17–0.59)	<0.001
Shortness of breath	0.58	(0.36–0.91)	0.018
Normal lung examination	0.58	(0.38–0.89)	0.013
			
**ED antibiotics within 4 hours**			
Age ≥65	1.1	(0.83–1.43)	0.484
Cerebrovascular disease	1.87	(1.12–3.11)	0.016
Temperature†	1.11	(1.04–1.19)	0.003
Respiratory rate†	1.05	(1.02–1.07)	<0.001
Normal lung examination	0.59	(0.43–0.81)	0.001
White blood cell count†	1.02	(1.001–1.04)	0.038
Chest x-ray with pneumonia	1.74	(1.31–2.30)	<0.001
			
**Blood cultures in ED**			
Age ≥65	1.02	(0.78–1.32)	0.9
Cough	1.31	(1.01–1.70)	0.04
Fever	1.68	(1.28–2.22)	<0.001
Abdominal pain	2.26	(1.27–4.03)	0.006
Confusion	2.03	(1.34–3.07)	0.001
Neoplasm	1.85	(1.35–2.53)	<0.001
Liver disease	3.02	(1.30–6.99)	0.01
Temperature†	1.16	(1.08–1.24)	<0.001
Systolic blood pressure†	0.99	(0.99–1.00)	0.007
Chest x-ray with pneumonia	1.38	(1.06–1.79)	0.018
			
**Assessment of oxygenation in ED**			
Age ≥65	2.10	(1.18–3.72)	0.011
Shortness of breath	2.27	(1.37–3.75)	0.001
Heart rate†	1.02	(1.004–1.03)	0.012
Fever	0.53	(0.31–0.89)	0.016
Abdominal pain	0.45	(0.21–0.96)	0.039

* Values are odds ratios (95% CI). P values represent the Wald statistic.

†Continuous variables

Age did not have an impact on the odds of receiving antibiotics in the ED within four hours (OR 1.10, 95%CI 0.84–1.43). An x-ray consistent with pneumonia, an elevated temperature at triage, increased respiratory rate, elevated white blood cell count, and a history of cerebrovascular disease were all associated with increased likelihood of receiving antibiotics within 4 hours. Two hundred forty three subjects were excluded from this model due to missing data within continuous variables. No interaction terms had a significant impact on this model.

Likewise, age was not a contributing factor in the decision to obtain blood cultures (OR 1.02, 95%CI 0.78 – 1.32). This rather large model indicates that multiple patient characteristics go into the decision to obtain blood cultures; after adjusting for these covariates, age ≤ 65 did not appear to be one of them. Blood cultures were more often performed in patients with liver disease, neoplasm, several presenting complaints (cough, fever, abdominal pain, and confusion), an x-ray consistent with pneumonia, or with initial elevated temperature. An interaction between a history of asthma and corticosteroid use turned out to be a nearly perfect predictor of blood culture utilization; however, the model with this interaction term included was highly unstable with large standard errors. Thus, the main effects model was retained. Thirty nine subjects were excluded due to missing data regarding triage temperature (2), systolic blood pressure (28), or both (9).

Age ≥ 65 was associated with increased odds of having oxygenation assessed in the ED (OR 2.10, 95% CI 1.18–3.72). Subjective complaints of fever or abdominal pain decreased the odds of oxygenation assessment, while increased heart rate and a complaint of shortness of breath increased odds of oxygenation assessment. Two interaction terms (steroid use × asthma and steroid use × COPD) were perfect predictors of oxygenation assessment and were thus unsuitable for inclusion; no other interaction terms were significant. Nine subjects were excluded due to missing data on heart rate at triage.

ANOVA was used to determine whether processes of care varied between age group subdivisions in the portion of the sample with age ≥ 65 (Table 3). Division of those ≥65 years of age into groups by decade revealed no differences in the percentage receiving any process of care in any decade.

**Table 3:** Percentage of Patients Receiving Each Process of Care Stratified by Age*

	**Age <65 years**		**Age ≥65 years compared by decade**						
			Age 65–74 years		Age 75–84 years		Age ≥85 years		p value by decade ≥65 years
**Process:**	(n = 810)		(n = 262)		(n = 209)		(n = 89)		
ED antibiotic administration	89.4	(86.8–94.1)	94.3	(91.1–96.9)	94.3	(90.9–97.1)	93.3	(87.7–98.3)	0.941
ED antibiotics within 4 hours	65.3	(61.7–68.3)	67.9	(62.3–73.7)	67.0	(60.5–73.5)	70.8	(61.6–80.4)	0.816
ED blood cultures	68.9	(65.9–72.1)	65.6	(60.3–71.7)	68.4	(61.7–74.3)	74.2	(65.0–83.0)	0.335
ED oxygenation assessment	94.0	(92.4–95.6)	95.8	(93.6–98.4)	96.6	(94.6–99.4)	98.9	(96.8–100)	0.488

*Patients who received pre-ED antibiotics excluded. Numbers are percent (95% CI)

## Discussion

The objective of this study was to determine if elderly ED patients admitted with pneumonia were less likely to receive recommended care interventions than younger patients. We found no relative deficiencies in care received, and actually found that elders were more likely to receive antibiotics and to have oxygenation assessed in the ED. The rate of process implementation did not vary by decade over age 65.

The Pneumonia National Quality Measures are used by CMS and JCAHO as well as private entities to assess quality of care for patients with pneumonia [7]. The requirement for rapid administration of antibiotics is supported by several observational studies which demonstrated decreased mortality in patients with rapid (<4–8 hours) time to antibiotics [11,12,15]. Elders may benefit from timely antibiotics even more than other populations [16]. Recommendations for oxygenation assessment are based on the observation that hypoxemia is associated with increased mortality [11].

However, controversy has arisen regarding the usefulness of individual measures, particular time to antibiotics and need for blood cultures [10,11,16,17]. The blood culture quality measure has been recently modified to include cultures only for those patients going to an intensive care unit within 24 hours of arrival, and to only require blood cultures prior to antibiotics if drawn in the ED [7]. It is based on specialty society recommendations [18] as well studies that have demonstrated an association between blood culture collection and decreased mortality [11]. This measure has been criticized by other studies demonstrating low rates of true positive cultures, equivalent false-positive rates, and rare changes in management [17].

Despite these concerns, monitoring of adherence to Pneumonia Quality Measures is now widespread. However, these recommended processes of care have not been uniformly applied to all patient populations. For example, a 2004 study by Mortensen *et al *found that black patients were less likely to receive antibiotics within eight hours of presentation than whites, but were just as likely as whites to have blood cultures obtained, to have oxygenation assessed, and to receive guideline-concordant antibiotics [8]. Similar disparities have also been documented in inpatients with pneumonia [5,19,20]. Of note, we did not any significant management differences based on race within our sample.

The elderly are another group at potential risk of disparities in care. Studies of other disease processes in the ED have found that age can influence the quality of care a patient receives. In 2005, Magid *et al *found that elderly patients presenting to the ED with myocardial infarction were significantly less likely than younger patients to receive aspirin, beta-blockers, and reperfusion therapy. This occurred even when there was no contraindication to the therapy [9]. This work confirmed studies with similar results in elder inpatients [21-23].

In elders with pneumonia, Fine *et al *in 2002 found that a significant percentage of patients do not receive optimal care [5]. They studied patient and hospital characteristics associated with care processes in elderly Medicare patients hospitalized with pneumonia. Several factors were associated with failure of process including nonwhite race, hospital teaching status and size, and hospital location in the South. Presence of fever was positively associated with process performance, as we found with certain measures (antibiotics within 4 hours and blood cultures) in this study. However, the applicability of Fine's study to current ED practice is unclear as the data was obtained in 1994–1995, only 57% of study patients were admitted through the ED, and guidelines at that time did not emphasize prompt antibiotic therapy. Furthermore, it did not investigate potential disparities between elders and younger patients.

Waterer *et al *described delayed administration of antibiotics in 451 patients with community-acquired pneumonia [10]. In this study, age when assessed as a continuous variable was minimally associated with delay in antibiotic administration (OR 1.01; 95% CI, 1.00–1.06). Delay in treatment was more strongly associated with a nonspecific clinical presentation including presence of altered mental state (OR 2.89; 1.53–5.45), absence of hypoxia (OR 1.82; 1.09–3.04), and absence of fever (OR 1.59; 1.06–2.40). All are factors associated with the presentation of pneumonia in elders.

This previous work demonstrates several potential reasons that diagnostic and therapeutic interventions may be delayed in elders. These include preconceived physician biases towards the group, concerns over side effects or complications of therapy, and delays in diagnosis due to nonspecific presentation [9]. The results of this study which reveal that elders do not suffer from inadequate process of care administration in the ED alleviate these concerns. In our institution, elders received equal or more aggressive care as younger patients.

We suspect that concern over potential side effects of therapy would most commonly be found in patients undergoing invasive procedures or receiving medications with potentially severe side effects (e.g., thrombolytics). We speculate that in the case of pneumonia, the non-invasive nature of obtaining blood cultures and assessing oxygenation as well as the relative lack of direct contraindication to most antibiotic therapies prevented concerns over side effects from contributing to a difference in care patterns.

Several studies have demonstrated that elders with pneumonia are less likely to develop common clinical symptoms, including a >50% reduction in the rate of fever as compared to patients <65 years of age [24,25]. In our database, elders were less likely to present with classic symptoms of pneumonia such as cough, fever, and shortness of breath. They were more likely to present with the non-specific complaint of confusion. We identified an independent association between antibiotic administration and confusion as antibiotics were less likely to be given in patients with confusion. This finding is consistent with that of Waterer *et al *who also found that an altered mental state was an independent predictor of failure to receive antibiotics within 4 hours (OR 3.2; 95% CI, 1.4 to 6.1). However, in aggregate their non-specific clinical presentation did not prevent elders from receiving appropriate processes of care as compared to the younger population. Similarly in elders with myocardial infarction, Magid *et al *found that differences in presentation alone did not explain process of care differences in elderly versus younger patients.

Although as a group elders were actually more likely to receive antibiotics, the data from the regression analysis provides information on areas to target for further improvement. Patients (both elder and non-elder) who are confused, have a normal lung exam, have HIV, or complain of shortness of breath are less likely to receive antibiotics in the ED. Those with lower temperature, lower respiratory rate, or normal lung exam are less likely to receive antibiotics within 4 hours. Greater sensitivity on the part of the physician to the diagnostic difficulties inherent in non-specific clinical presentations should result in performance improvement on the quality measures, particularly early antibiotic administration.

The patient's PSI score was not included in the regression models for two reasons. First, it covaries strongly with age (r = 0.60). Second, it has been suggested to be inaccurate in the elderly and thus evaluation of individual variables was warranted[26,27]. As an alternative, we included most of the components of the PSI as individual components in the regression.

A primary limitation of the study was its retrospective chart review nature. However, steps were taken to ensure accuracy of data abstraction, a large percentage of data elements were completed, and agreement of the abstractors was good for multiple variable types (process, history, symptoms, laboratory, vital signs). Data was collected from only one center and results may not be easily generalized to all EDs, particularly as individual hospital characteristics have been shown to influence performance of these measures [5]. However, our findings demonstrate equivalent or better process implementation as compared to previous studies [5]. To provide conservative estimates, missing data for process measurements was coded as not performed. We are confident that there is good concordance for each of the primary study outcomes. It is likely that the antibiotics were actually administered in each case as it would be rare for a nurse to chart a specific administration time without administering the medication. Likewise, actual values are recorded for oxygen assessment. It is unlikely that such values were fictitious. For blood cultures, we attempted to ensure that they were actually obtained by requiring not just a physician order but also either documentation by the nurse or documentation of receipt in the microbiology lab.

We chose to include all patients who received a diagnosis of pneumonia in the ED [28]. Strict interpretation of the JCAHO criteria would result in exclusion of patients who do not have an abnormal chest x-ray or CT scan during the hospitalization. We found that 15% of our study patients did not have an abnormal chest x-ray in the ED, but due to database limitations we were unable to account for rates of chest x-ray negative, CT-scan positive pneumonia. Such rates have generally been approximately 7% in ED patients with pneumonia [28,29]. We are confident that the inclusion of patients without chest x-ray abnormality but with ED diagnosis of pneumonia did not introduce substantial error into our findings for several reasons. First, such an approach has previously been used in the literature, and rates of patients failing to meet strict JCAHO inclusion criteria are no higher in our study [28]. Also, in our study the rates of antibiotic administration were the same between those with and without abnormal chest x-rays. Based on past work, we expect that approximately 7% of ED patients will have negative chest x-rays but positive CT scans [28,29]. In addition, patients with abnormal chest imaging during their admission also meet JCAHO criteria. As a result, the true rate of patients without any abnormality on imaging study should be much lower than 15%. Given that ED physician behaviour is based on clinical diagnosis at the time of patient interaction rather than post-hoc radiologic interpretation, we feel that this methodology provides the most accurate measure of behaviour.

Among the recommended process measures, we chose not to assess influenza vaccination, pneumonia vaccination, or smoking counseling as these are generally performed on the inpatient wards. We also did not address antibiotic appropriateness as recommended antibiotics vary depending on several factors including presence of healthcare-associated pneumonia, structural lung disease, recent antibiotic use, or an immunocompromised state [18,30]. Due to the nature of our database, we were unable to guarantee accurate determination of several of these variables and therefore could not judge appropriateness of antibiotic selection. The study also only examined ED course and did not account for meeting the process of care requirements after admission. We believe this would likely increase compliance with the blood culture and oxygen measurement guidelines. Combining the time spent in the ED with the delays in initiating antibiotics after admission, we do not believe it would change significantly the proportion of patients receiving antibiotics within 4 hours.

The study may suffer from incorporation bias, which in this case could occur when the variable being measured, advanced age, is one that could also affect both the diagnostic and treatment decisions leading to enrollment in the study. The presence of such bias could affect the final results and effect measures of the study.

In this study, such bias could occur due to differences in admission decision and diagnostic evaluation between elders and non-elders. The potential for and magnitude of admission bias can be seen in the absolute rates of admission, 60.1% for younger patients and 88.0% for older patients. Additional potential incorporation biases could be due to age-related differences in pursuing and making the diagnosis itself. Such an effect is difficult to measure for several reasons. For example, as elderly patients often present with non-specific signs and symptoms of pneumonia, the use of symptom-based inclusion criteria would result in significant spectrum bias. Thus to identify diagnostic accuracy in patients would require examination of the entire population of ED elderly patients, regardless of presenting symptom. We chose to use the previous literature as a guide and chose methods similar to other studies for inclusion criteria. The fact that rates of radiographic evidence of pneumonia were similar between groups does provide some assurance regarding the specificity of the diagnosis, although it does not provide information about diagnostic sensitivity. As a result of these issues, in this retrospective study we were unable to confirm accuracy of diagnosis, adequacy of diagnostic workup, or reasoning behind the admission decision. All of these factors affected patient entry into the study and potentially contributed to incorporation bias.

The effect of incorporation bias is somewhat mitigated by the fact that our process outcome measures are not directly related study entry. That is, these process measures are not directly related to the decision to make an initial diagnosis of pneumonia or admit the patient. The most egregious example of incorporation bias would be using clinical manifestations to make a diagnosis and then examining the frequency of those clinical findings in the patients so diagnosed. In this study, the mere act of administering antibiotics, obtaining blood cultures, or checking pulse oximetry is not generally a component of making a pneumonia diagnosis or admission decision and thus not part of triggering study entry criteria. Although obtaining a pulse oximetry may more often be done in patients suspected of pneumonia, its ubiquitous use means that the obtaining of pulse oximetry (as distinct from the value obtained) is not a criterion for pneumonia diagnosis. However, this does not mitigate the bias discussed above which may be introduced due to difference in admission and diagnosis decisions between patients of different ages and so it must be stressed that our study only applies to a patient population constituted after a diagnosis and admission decision is made.

There is also a possibility that the differential admission rates between elders and younger patients may have arisen due to different admitting paradigms on the part of ED physicians with the result that comparison between the two groups is meaningless. For example, younger patients may have been admitted because they appeared to the ED physician to be more ill, while elder patients may have had a greater weight given to age alone. Such imbalance likely represents real-world practice, as even the PSI places a large weight on age and co-morbidities. Although we cannot specifically measure the effect that such differences would have, we do provide information regarding differences in populations in Table 1. The populations do differ in symptoms at presentation and co-morbidities. However, there are no differences in rates of abnormal vital signs or chest x-rays. We then performed a regression analysis to determine if age was truly a factor independently affecting process (Table 2). It should be cautioned that, although these methods do provide a comparison between groups and examine age as an independent variable, they were only measured in patients meeting study entry criteria so that any interpretation must still consider potential incorporation bias as discussed above.

The result of this heterogeneity between populations is that the study is limited in several ways. We are unable to determine why a particular process measure was or was not followed. We can only provide the rate of process conformity. We can only conclude that in the population of patients diagnosed and admitted with pneumonia, the rates of compliance with recommended processes of care are as noted and are affected by the covariates noted in the regression model. This ultimately limits the generalizability of our study results as we cannot make inferences on the data we did not analyze. The results apply only to those patients diagnosed with pneumonia and in whom the decision is made to admit. They do not apply to the infected elderly population in general or to patients prior to the diagnosis or admission decision.

## Conclusion

In conclusion, this study attempted to determine if the level of care accorded to older ED patients admitted with a diagnosis of pneumonia was consistent with that accorded to younger ones, as measured by the Pneumonia National Quality Measures. We found that elderly patients diagnosed with pneumonia receive equal or better quality care in the ED than younger patients. There does not appear to be a difference based on age over 65 by decade. There is an independent association of certain patient characteristics such as confusion, absence of fever, and a normal lung exam with process failure. This provides an opportunity to further increase compliance with the quality measures by identifying patients at risk of process failure.

## Competing interests

The authors declare that they have no competing interests.

## Authors' contributions

JMC developed the hypotheses, analyzed the database, and wrote the manuscript. BCH participated in the design of the study, performed the statistical analysis, and edited the manuscript. DRM supervised the creation and collection of the database, participated in the design of the study, and edited the manuscript. All authors read an approved the final manuscript.

## Pre-publication history

The pre-publication history for this paper can be accessed here:


